# Mining potentially actionable kinase gene fusions in cancer cell lines with the KuNG FU database

**DOI:** 10.1038/s41597-020-00761-2

**Published:** 2020-11-30

**Authors:** Alessio Somaschini, Sebastiano Di Bella, Carlo Cusi, Laura Raddrizzani, Antonella Leone, Giovanni Carapezza, Tommaso Mazza, Antonella Isacchi, Roberta Bosotti

**Affiliations:** 1 NMS Oncology, Nerviano Medical Sciences, NMS Group, 20014 Nerviano, Milan Italy; 2 Bioinformatics Unit, IRCCS “Casa Sollievo della Sofferenza”, Research Hospital, San Giovanni Rotondo, Italy

**Keywords:** Cancer genomics, Target identification

## Abstract

Inhibition of kinase gene fusions (KGFs) has proven successful in cancer treatment and continues to represent an attractive research area, due to kinase druggability and clinical validation. Indeed, literature and public databases report a remarkable number of KGFs as potential drug targets, often identified by *in vitro* characterization of tumor cell line models and confirmed also in clinical samples. However, KGF molecular and experimental information can sometimes be sparse and partially overlapping, suggesting the need for a specific annotation database of KGFs, conveniently condensing all the molecular details that can support targeted drug development pipelines and diagnostic approaches. Here, we describe KuNG FU (KiNase Gene FUsion), a manually curated database collecting detailed annotations on KGFs that were identified and experimentally validated in human cancer cell lines from multiple sources, exclusively focusing on in-frame KGF events retaining an intact kinase domain, representing potentially active driver kinase targets. To our knowledge, KuNG FU represents to date the largest freely accessible homogeneous and curated database of kinase gene fusions in cell line models.

## Introduction

Genomic instability is one of the hallmarks of cancer^1^. The occurrence of complex chromosomal rearrangements, such as inversions or translocations, can result in novel chimeric fusion genes, potentially representing driver events in cancer development^2,3^. Fusion genes were the focus of recent systematic analyses of Next-Generation Sequencing (NGS) transcriptomic datasets: Picco and colleagues reported the prediction of 7,430 unique gene fusions in more than 1,000 human cancer cell lines, across 42 different tissue types^4^; Gao and colleagues identified over 25,000 fusion transcripts, revealing 1,275 KGFs involving an intact catalytic domain in almost 10,000 clinical tumor samples, representing 33 cancer types in The Cancer Genome Atlas (TCGA)^5,6^. In this analysis, an overall low gene expression level was described for tumor suppressor gene fusions. At the same time, high gene expression was observed for fusions involving kinases and oncogenes, with the latter representing appealing druggable targets by approved or newly designed anticancer compounds^6^.

As of today, there are about 50 kinase inhibitor drugs approved by FDA in Oncology^7^, the majority of which are Tyrosine Kinase inhibitors. The first example of a successful KGF targeted therapy is imatinib (Gleevec, Novartis), specifically active on a BCR-ABL1 kinase fusion in chronic myeloid leukemia patients^8^. Later on, more targeted drugs have been developed against well-recognized driver kinases activated in cancer due to KGFs, such as those involving ALK, RET, ROS1, NTRK or FGFR3^9^. Remarkably, two drugs targeting KGF-activated NTRK kinases, namely entrectinib (Rozlytrek, Roche), also approved for the treatment of ROS1-positive patients with metastatic non-small cell lung cancer (NSCLC)^10,11^, and larotrectinib (Vitrakvi, Bayer^12^), got accelerated FDA approval as the first tissue-agnostic drugs for the treatment of tumors testing positive for NTRK kinase fusions, regardless of the cancer type, shedding further light on the importance of KGFs as cancer targets.

Precision medicine strongly relies on research models^13,14^ and cancer cell lines can represent widely accessible tools for the investigation and the functional characterization of therapeutically actionable KGF targets, paralleling the genetic alterations present in primary tumor samples^11,15–19^. Extensive KGF molecular annotation would therefore greatly support the drug development pipeline and aid the parallel advancement of companion diagnostic tools for the assessment of patient treatment eligibility, such as multiplex amplicon approaches^20^ or Anchored Multiplex PCR (AMP)^21^.

Public databases collecting newly NGS-identified gene fusions are increasingly available, as listed by Gioiosa and colleagues^22^; among these, the major sources of predicted KGFs in human cancer cell lines are the work by *Klijn et al*.^13^ and the LiGeA DB^22^. Klijn and colleagues have collected over 2,200 gene fusions resulting from the bioinformatics analysis of RNA-seq data through multiple fusion calling algorithms; however, experimental assessment of the oncogenic KGF functional role is not provided and the specific accession IDs of the transcripts involved in the fusion events are not specified. Gene fusions are reported as plain text lists, preventing interactive queries. The LiGeA database is a searchable and interactive resource of gene fusions in human cancer cell lines, containing ~1700 *in silico* predicted fusions identified by reanalyzing RNA-sequencing experiments performed by the Cancer Cell Line Encyclopedia (CCLE^23^), using four different gene fusion detection algorithms^22^. Though more practical, this database still suffers from the lack of experimental validation information and from incomplete sequence annotation, such as the exact breakpoints or the transcript IDs of the genes involved in the fusion events; moreover, no coverage is provided for KGFs identified in cell lines not included in the CCLE dataset. Indeed, a considerable number of human cancer cell lines with characterized and functionally validated KGFs are described in sparse literature, but are not included in any collective repository available for this kind of information.

To overcome these limitations, we have implemented the KuNG FU (KiNase Gene FUsion) database, a manually curated repository of KGFs in cancer cell lines, collecting comprehensive and detailed annotations of in-frame KGF events retaining an intact kinase domain sequence and for which experimental evidence has been reported. The above selection criteria represent fundamental KGF prerequisites for potential druggability investigations, making KuNG FU the largest homogeneous and curated database of KGFs in cell line models available to date (open access at http://www.kungfudb.org/).

## Results

### KuNG FU overview

**Fig. 1:**
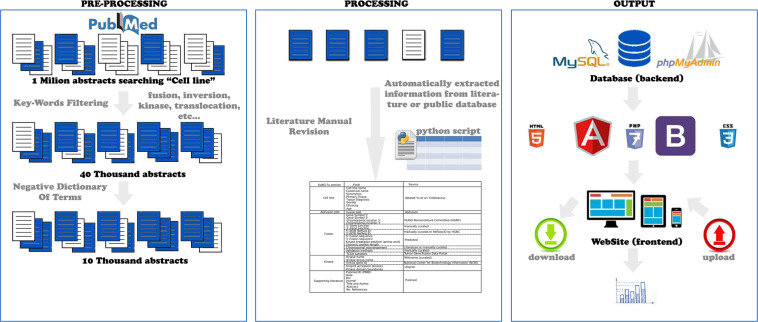
KuNG FU Workflow. Schematic illustration of the workflow followed for the implementation and feeding of the KuNG FU database: automated and manually curated ‘Pre-Processing’ and ‘Processing’; database structure and interface construction (‘Output’). See detailed description in M&M section.

We started the KuNG FU database implementation by collecting relevant data through automated searches followed by extensive manual curation (Pre-Processing and Processing), for the extraction of KGF information obtained from the data mining of over a million scientific abstracts, dated starting from 2013 and integrated with public datasets and previous literature, as summarized in the schema reported in Fig. 1 and described in detail in the M&M section. The resulting dataset was stored in a MySQL database (Fig. 1, Output).

KuNG FU data access is equipped with a search engine that supports free-text searches, as well as filtering based on keywords, through an intuitive web interface, allowing researchers to query among a broad panel of human cancer cell lines and providing graphical summaries of the database content (Fig. 1, Output). Remarkably, over half of the KuNG FU database includes cell lines carrying potentially actionable KGFs derived from sparse literature and not included in the CCLE or in any other databases that were interrogated during the Processing phases, thus highlighting the added value of the KuNG FU database^13,22–25^.

**Fig. 2:**
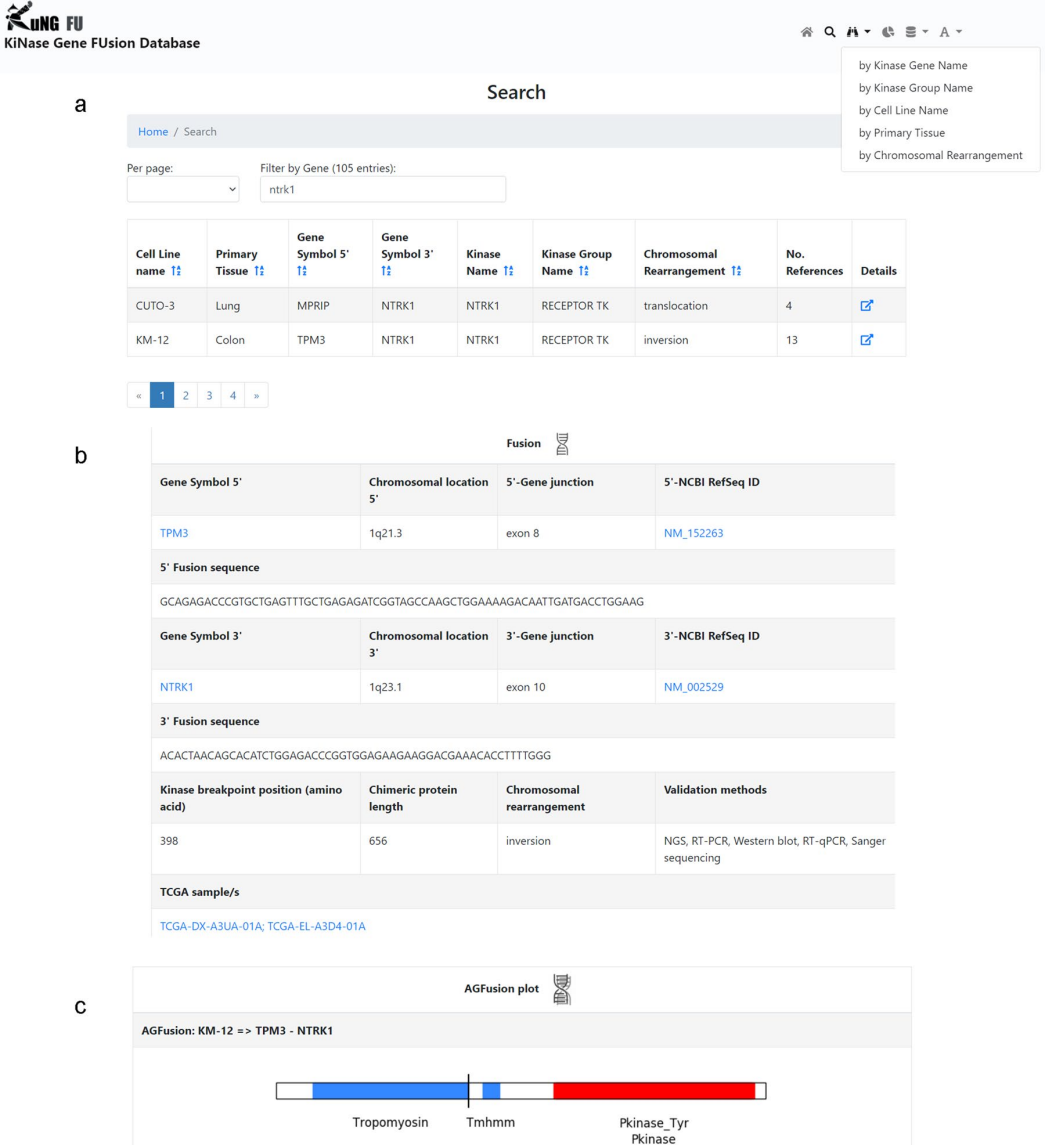
KuNG FU user interface. Representative screenshots of the KuNG FU user interface showing an example query for the NTRK1 gene. **(a)** KuNG FU query module (top) and summary table (bottom). The query type can be selected from the scroll-down menu or typed in the ‘Filter’ box; the query immediately provides a summary table with links to the ‘Details’ sections for each KGF found in the database. **(b)** ‘Fusion’ sub-section, reporting the chromosomal coordinates and the RefSeq transcript IDs of the kinase gene and its respective fusion partner, together with the exons/introns involved in the fusion and the chimeric transcript nucleotide sequence at the breakpoint, with details about the position of the predicted breakpoint in the kinase, the predicted length of the resulting chimeric protein, the type of chromosomal rearrangement event and the experimental methods supporting the KGF. For KGFs that were also identified in clinical samples, links to TCGA patient ID/s in Tumor Fusion Gene Data Portal^27^ are provided **(c)** AGFusion plot section, displaying a diagram of the protein domain architecture of the queried chimeric fusion transcript generated with the AGFusion plot tool^26^.

The KuNG FU query output (example screenshots in Fig. 2) contains all the data types collected for each KGF, organized in sub-sections, such as ‘Cell Line’, ‘AGFusion Plot’, ‘Fusion’, ‘Kinase’ and ‘Supporting Literature’ annotation fields, as listed in detail in Table 1 with the respective data sources.

**Table 1:** KuNG FU database fields.

KuNG Fu section	Field	Source
**Cell line**	**Cell line name**	dataset *Yu et al*./Cellosaurus
	**Canonical name**	dataset *Yu et al*./Cellosaurus
	**Synonym/s**	dataset *Yu et al*./Cellosaurus
	**Primary Tissue**	dataset *Yu et al*./Cellosaurus
	**Tissue Diagnosis**	dataset *Yu et al*./Cellosaurus
	**Gender**	dataset *Yu et al*./Cellosaurus
	**Ethnicity**	dataset *Yu et al*./Cellosaurus
	**Age**	dataset *Yu et al*./Cellosaurus
**AGFusion plot**	**Fusion plot**	AGFusion^26^
**Fusion**	**Gene Symbol 5**′	HUGO Gene Nomenclature Committee (HGNC)
	**Gene Symbol 3**′	HUGO Gene Nomenclature Committee (HGNC)
	**Chromosomal location 5**′	HUGO Gene Nomenclature Committee (HGNC)
	**Chromosomal location 3**′	HUGO Gene Nomenclature Committee (HGNC)
	**5**′**- Gene junction**	manually curated
	**3**′**- Gene junction**	manually curated
	**5**′**- NCBI RefSeq ID**	manually curated or RefSeq ID by HGNC
	**3**′**- NCBI RefSeq ID**	manually curated or RefSeq ID by HGNC
	**5**′**- Fusion sequence**	Predicted
	**3**′**- Fusion sequence**	Predicted
	**Kinase breakpoint position (aa)**	Predicted
	**Chimeric protein length**	Predicted
	**Chromosomal rearrangement**	Literature or manually curated
	**Validation methods**	Manually curated
	**TCGA sample/s**	Tumor Fusion Gene Data Portal
**Kinase**	**Kinase name**	Wikinome (curated)
	**Kinase group name**	Wikinome (curated)
	**Entrez gene ID**	National Center for Biotechnology Information (NCBI)
	**Uniprot accession (kinase)**	Uniprot
	**Kinase domain boundaries**	Uniprot
**Supporting literature**	**Pubmed ID (PMID)**	Pubmed
	**Date**	Pubmed
	**Doi**	Pubmed
	**Journal**	Pubmed
	**Title and Author**	Pubmed
	**Abstract**	Pubmed
	**No. References**	Pubmed

Summary of fields and associated sources included in each section of the KuNG FU database.

Users can interrogate KuNG FU by means of kinase gene name, kinase group name, cell line name, primary tissue or by chromosomal rearrangement event generating the KGF (Fig. 2a, top). Upon filtering, a summary table is provided for the selected KGFs, reporting cell line, tissue and molecular details such as fusion partner and chromosomal rearrangement event (Fig. 2a, bottom). By clicking on the “Details” tab, for each KGF an output page is displayed, organized in sub-sections, such as ‘Cell line’, ‘Fusion’ (Fig. 2b), ‘AGFusion plot’ (Fig. 2c), ‘Kinase’ and ‘Supporting literature’ (Table 1). These sub-sections provide detailed KGF molecular information, such as breakpoints and respective specific transcripts and introns/exons involved in the fusion event (Fig. 2b), along with the AGFusion plot^26^, displaying a graphical representation of the KGF construct and protein domains for visual inspection (Fig. 2c).

Each KGF annotation dataset also includes information on published experimental methods used for KGF validation with several supporting literature references and links to the Tumor Fusion Gene Data Portal database^27^ for corresponding KGF events detected in patient-derived tumor samples. Only in-frame KGFs retaining an intact catalytic domain were included in KuNG FU, to offer a selected set of targets characterized in cancer cell line models, possessing the necessary sequence prerequisites for potential KGF druggability. These prerequisites are definitely met in the KGFs targeted by drugs in the clinics^11,28–32^. KuNG FU also provides a ‘Statistics’ section allowing a graphical representation of the database content, in particular showing the distribution of kinase groups, tissues of origin for cell lines, and types of aberrant chromosomal events that occurred to generate the KGFs. The KuNG FU data content at the moment of manuscript finalization is open to regular yearly updates and can be exported as txt files; moreover, users are encouraged to contribute with their own experimentally validated KGF data through a web based submission form, by visiting the Upload Page on the KuNG FU website http://www.kungfudb.org/upload.php.

### KuNG FU statistics

**Fig. 3:**
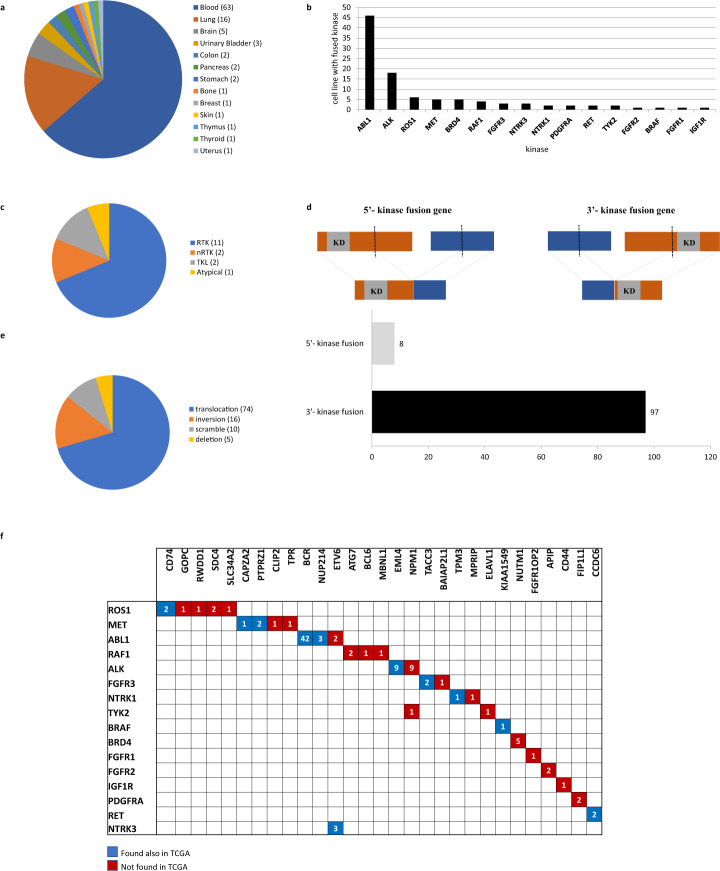
KuNG FU statistics and KGFs. Graphical representation of the distribution of the KuNG FU database content. **(a)** Cell line tissue of origin; **(b)** Number of cell lines harboring a specific kinase involved in a KGF; **(c)** Kinase groups (RTK = Receptor Tyrosine Kinase, nRTK = non- Receptor Tyrosine Kinase, TKL = Tyrosine Kinase-Like); **(d)** 5′- and 3′- kinase fusions; **(e)** Type of aberrant chromosomal event occurred (‘scramble’: complex genetic event, as in *Klijn et al*.^13^); **(f)** Matrix showing the combination types of the 16 kinases (left) with the associated fusion gene partners (top) for the KGFs included in KuNG FU, color-coded by detection in cell lines only (red) or both in cell lines and TCGA samples (blue). For each kinase/gene partner combination, numbers in blue and red boxes indicate the total number of individual KGFs currently reported in KuNG FU.

In the current release, the KuNG FU database contains 105 in-frame KGF events retaining an intact catalytic domain, supported by published experimental sequence evidence. These KGFs belong to 101 different human cancer cell lines; intriguingly, over half of these were not previously listed in any collective cell line databases reporting validated KGFs, such as CCLE^22–24^, *Kljin et al*.^13^ or the Cell Line Project resource^25^, but were retrieved from individual publications, highlighting the novelty of the KuNG FU unique database content. Overall, cell lines in KuNG FU are derived from 13 different tumor types, among which the majority are of hematologic origin, while solid tumors are enriched in lung or brain cancer derived cell lines (Fig. 3a). The 105 experimental validated fusion events collected in KuNG FU involve 16 different kinases (Fig. 3b).

A substantial enrichment in kinases belonging to the Tyrosine Kinase (TK) group is observed, in particular for Receptor Tyrosine Kinases (RTKs). The remaining gene fusion kinases reported in KuNG FU belong to Tyrosine Kinase-Like (TKL) and Atypical groups (Fig. 3c). Moreover, a clear enrichment of fusions harboring the kinase catalytic domain at the 3′-end of the gene is observed (92%). Only the 8% of the KGFs rearrange with the kinase domain at the 5′-end (Fig. 3d), in agreement with previously reported observations in clinical samples^3,33^. Chromosomal rearrangement events represented in KuNG FU are translocations, inversions, deletions or ‘scramble’ events, indicating complex genetic rearrangements, as in *Klijn et al*.^13^, with translocations being the most frequently observed aberrations (70%, Fig. 3e), in agreement with what reported for clinical samples^27^.

ABL1 is the most frequently rearranged kinase found in cancer cell lines, fused with different gene partners in 46 distinct cell lines (Fig. 3b), with the BCR-ABL1 fusion being the most frequent (Fig. 3f). Other frequently recurring fused kinases are ALK (found in 18 cell lines) and ROS1 (in 6 cell lines), both well known as cancer drivers in lung adenocarcinoma (EML4-ALK fusion) and non-small cell lung cancer (ROS1 rearrangements), for which different inhibitor drugs have been developed^34,35^. Almost all the fusion transcripts reported in KuNG FU are specific for a single cancer type, however three fusions (BRD4-NUTM1, CCDC6-RET and EML4-ALK) were found across a variety of different cancer types.

Eight out of the 16 kinases included in KuNG FU are rearranged with more than one gene partner, with ROS1 being the kinase with the largest fusion promiscuity (Fig. 3f). Emerging evidence suggests that the role of gene partners in kinase fusions is not limited to driving the expression of the chimeric transcript or its hyperactivation through oligomerization; gene partners could also be involved in the recruitment of other proteins, in the localization of the kinase in different cellular compartments or its regulation and stabilization^33,36^. Among the 29 identified fusion partners, BCR is the most frequently observed in cancer cell lines (Fig. 3f). Interestingly, only ETV6 and NPM1 are found as promiscuous partners, while the other 27 gene partners are always found in fusion events with a single specific kinase (Fig. 3f), in agreement with previous studies^37^.

Alternative breakpoint usage is observed in the rearrangements of 9 kinases. Interestingly, ROS1 and BRD4 KGFs occur at four different breakpoint sites; conversely, ALK and RET are found rearranged using the same breakpoint in different cancer cell line models, regardless of the cancer type context (Table 2). Intriguingly, alternative breakpoint usage generating multiple KGFs can also be found within the same cell line, as in CUTO-2 and SNU-16, where the same portion of the partner gene (SDC4 or APIP, respectively) is fused with 2 different breakpoints of the same kinase (ROS1 or FGFR2, respectively), or in ALL-VG, where two alternative breakpoints in the ETV6 partner gene generate 2 different KGFs with the same ABL1 kinase portion. In these instances, the summary table of the KuNG FU query results will list two distinct KGF entries for the same cell line.

**Table 2:** KuNG FU kinase breakpoint sites.

kinase	n° of breakpoint sites	breakpoint (aa)
**BRD4**	4	682 (lung), 719 (lung/NMC), 1056 (thymus/NMC), 1093 (NMC)
**ROS1**	4	991 (breast), 1749 (lung), 1852 (lung), 1880 (brain)
**MET**	3	5′-UTR (brain), 620 (uterus), 1009 (brain/bone)
**RAF1**	3	141 (blood), 226 (pancreas), 278 (pancreas/brain)
**FGFR2**	2	208 (stomach), 429 (stomach)
**FGFR3**	2	759 (urinary bladder), intron 18 (urinary bladder)
**NTRK1**	2	398 (colon), 451 (lung)
**NTRK3**	2	465 (blood), 528 (blood)
**TYK2**	2	725 (skin), 872 (blood)
**ABL1**	1	26 (blood)
**ALK**	1	1057 (blood, colon, lung)
**BRAF**	1	380 (brain)
**FGFR1**	1	428 (blood)
**IGF1R**	1	828 (stomach)
**PDGFRA**	1	551 (blood)
**RET**	1	712 (lung, thyroid)

Number of KGF breakpoint sites for each kinase included in the KuNG FU database. In brackets, amino acid (aa) position of the breakpoint and tumor tissue type where the breakpoints occur.

The overlap between KGFs detected in tumor cell lines and clinical cancer samples from TCGA showed that 65% of KGFs represented in KuNG FU are also present in TCGA clinical samples (Fig. 3f). This suggests that cell line models can recapitulate patient tumors, as previously observed by Iorio and colleagues^38^, and can serve as surrogate systems for molecular mechanism and drug efficacy investigations^11,15^.

## Discussion

In-frame KGFs bearing an intact kinase domain can be highly tumorigenic, altering the activity of the signaling pathways involved in cancer development^9^, and tumor cell lines represent easily accessible surrogate models that can be exploited to study the role of kinases and their susceptibility to inhibitors^38^. The identification of fusion candidates, which are likely to be biologically relevant^39^, was extraordinarily expanded by the systematic analysis of tumor samples by NGS approaches, together with the derivatization of tumor cell lines which can be grown *in vitro* and *in vivo* to test inhibitor sensitivity. However, NGS fusion detection algorithms still report a large number of false-positive candidates, suffering also from the lack of a sufficient set of experimentally validated KGFs, which could be used as positive controls for performance assessment of diagnostic methods^40^. In this scenario, the KuNG FU database was conceived to fulfill the need for an accurate database providing systematic annotations for experimentally validated KGFs in cancer cell lines. Multiple features account for the novelty of KuNG FU, from the specific and curated database content to the database ease of use, enabled by queries through a user-friendly web interface, allowing the quick interrogation of the database details for each KGF. Our conspicuous literature screening efforts (Fig. 1) resulted in a collection of 105 in-frame KGFs with an intact kinase domain, supported by published experimental evidence, found in 101 human cancer cell lines. This provides the richest centralized source of cell line models for KGF investigational studies, since only less than 50% of these cell lines were already included in CCLE^23,24^ or other collective sources reporting KGFs^13,22,25^; therefore, over half of the KGF-carrying cell lines listed in KuNG FU can be considered novel database content because they could otherwise only be retrieved from sparse literature. Curation of the 105 KGFs in KuNG FU was based on the assumption that an in-frame, intact kinase domain sequence within a KGF is likely to retain catalytic activity and conformational features prone to inhibition, as supported by numerous examples already targeted by compounds in the clinics^11,28–30,32,33^. Indeed, KGF events in cell lines often recapitulate molecular alterations found in clinical samples. Embedded cross-links to TCGA sample identifiers harboring the same kinase fusion provided by KuNG FU facilitate the interrogation of clinical datasets, in support to cell line model exploitation in all phases of drug discovery and development processes. In this respect, the enrichment in cell lines harboring similar or identical KGF events offers the possibility to investigate the same aberrant oncogenic event in the context of distinct complex molecular backgrounds, thus mimicking to a certain extent the patient-to-patient individual variability and providing a better functional understanding of different phenotypic effects, such as susceptibility to treatments, onset of resistance mutations or variable feasibility of *in-vivo* cell line engraftment models.

There are currently thousands of clinical trials testing the efficacy of novel kinase inhibitor drugs, and new molecules are approved every year^41,42^. All of these targeted therapies aim at a specific patient population that is exquisitely sensitive to the matched drug, which can be identified through cancer patients screening with a companion diagnostic able to detect the specific KGF. In this respect, the sequence details available in KuNG FU were specifically collected to allow further experimental KGF analysis and characterization. Therefore, KuNG FU might prove instrumental for the design of customized NGS panel for multiplex detection of fusion transcripts, which can be used in the clinic for patient population selection. Though not sufficient, the same kinase domain sequence integrity prerequisites applied in the selection of the KuNG FU KGFs are often the basis also for the development of companion diagnostic tools for KGF inhibitor treatment eligibility, searching for patients positivity to kinase rearrangements based on sequence assessment methods^30^, in some cases not even requiring prior knowledge of the involved partner gene^21^. Examples of such applications might be the design of probes for Fluorescence *In Situ* Hybridization (FISH) and RT-PCR methods^43^, or for multiplex amplicon and Anchored Multiplex PCR (AMP) NGS approaches^20,21,31,44^. These recently developed diagnostic strategies have some advantages, requiring low input, potentially increasing sensitivity associated to extensive amplification, short turnaround time and reducing the complexity of data analysis^31^.

Importantly, KuNG FU is freely accessible to the scientific community and is open to regular updates by literature checking for new validated KGFs on a yearly basis; additionally, it offers the unique feature for users to submit their validation experiments on KGFs, thus contributing to extending and maintaining the database up to date and making the tool a comprehensive useful resource for cancer investigators and drug research.

## Methods

### PRE-PROCESSING: literature and database automated mining

Abstracts published in PubMed between January 2013 and December 2019 were initially filtered using Python scripts for the presence of the keyword “cell line” appearing either in the Title or Abstract. The selected abstracts were then queried for the presence of the following keywords: “fusion”, “inversion”, “translocation”, “rearrangement” and “kinase” appearing either in the Title, Abstract or among the MeSH terms. A negative dictionary of terms was also created containing false positive terms to be excluded, such as “nuclear translocation”, “bacterial translocation”, “cell-cell fusion”, “membrane fusion”, etc.

### PROCESSING: literature manual revision and kinase gene fusion annotation

In-depth revision of the processed literature was applied for extensive manual curation of the identified KGFs. Reviewed information was integrated into the KuNG FU database schema.

All the fields listed in Table 1 were collected from the indicated sources and revised to provide detailed information, gathered in different sub-sections for ‘Cell Line’, ‘AGFusion plot’, ‘Fusion’, ‘Kinase’ and ‘Supporting Literature’ for each KGF. In particular, manually curated literature information was integrated with automatically extracted information from *Yu et al*.^45^, Cellosaurus database^46^, HUGO Gene Nomenclature Committee (HGNC) database^47^, Tumor Fusion Gene Data Portal^27^, WiKinome^48^, National Center for Biotechnology Information (NCBI)^49^ and UniProt^50^ websites through Python scripts. Three major fields describe the cell lines reported in KuNG FU: (1) ‘cell line name’, a unique name identifying the cell line; (2) ‘synonym/s’, cell line name synonyms (3) ‘primary tissue’, indicating the tissue from which the cancer cell was derived. Cell line names and synonyms were disambiguated by creating a list of unique identifiers extracted from *Yu et al*.^45^ and integrated with cell line synonyms from Cellosaurus^46^. Additional cell line description fields were included, reporting gender, ethnicity, patient age at diagnosis. Cross-contaminated cell lines, as per the register of misidentified cell lines curated by the International Cell Line Authentication Committee (ICLAC)^51^, were excluded from KuNG FU. In the ‘Fusion’ sub-section, HGNC^47^ approved gene symbols and RefSeq.^49^ transcript identifiers were used, with HGNC^47^ chromosomal coordinates of kinases and gene partners; 5′ and 3′ Gene Junction fields indicate the specific exon/intron involved in each breakpoint, and the ‘Chromosomal rearrangement’ field indicates the event generating the aberrant transcript (translocation, inversion, deletion or ‘scramble’^13^). ‘Validation methods’ is a manually curated field indicating the technical methodologies used for experimental KGF evidence found in the supporting literature along with the PubMed unique Identifier (PMID) for the respective reference(s). Additional wet-lab techniques used for experimental validation of the KGF sequence features were retrieved from 62 scientific papers published before January 2013 and integrated in KuNG FU, referenced with the corresponding PMID. TCGA^5^ identifiers of the samples harboring the same KGF found in KuNG FU cancer cell lines were also extracted and provided with a link to the TCGA Data Fusion Portal^27^. In the ‘Kinase’ section, for each kinase, NCBI^49^ Entrez Gene ID and UniProt^50^ protein accessions were provided, together with the ‘Kinase Group name’ and the ‘KD boundaries’ fields (KD: kinase domain).

The nucleotide sequence at the breakpoint, the amino acid breakpoint position in the kinase and the length of the resulting chimeric protein were also automatically calculated and reported in KuNG FU. When the transcript ID of kinase and fusion partner could not be retrieved from literature, fusion gene breakpoints were predicted based on the transcript RefSeq ID^49^ reported in HGNC^47^ that refers to a canonical UniProt^50^ sequence, chosen by criteria described in UniProt/Swiss-Prot website available at https://www.uniprot.org/help/canonical_and_isoforms. The conservation of the entire kinase domain within the KGFs and the observation of the reading frame in the resulting chimeric protein were calculated by means of the AGFusion tool^26^.

### OUTPUT: KuNG FU database and user interface

Collected data were stored in three MySQL relational tables: “cell_line_table”, storing meta information relative to cell lines; “abstract_table”, collecting the information extracted from the abstracts; “fusion_table”, reporting fields related to the KGF. MySQL database has been provided with a user-friendly graphical interface developed using AngularJS, a Javascript framework, and powered by JQuery and Bootstrap. Statistics are visualized through interactive graphs, implemented in PHP and Javascript. The fusion data stored in the database can be exported as txt and sql files, and a graphical representation of the gene fusion construct with respective protein domains is also provided through the AGFusion plot tool^26^ (Fig. 2c).

An application form allows users to submit experimentally validated kinase fusions not previously included in the database. Upon revision, data will be added to KuNG FU with the appropriate reference, if available, together with submitters’ acknowledgments.

## Data Availability

The curated database sql file and all python script files (Python version 3.6) used for abstracts retrieval from PubMed and for filling kinase gene fusion data are freely available on Zenodo (10.5281/zenodo.3996125)^52^.

## Code availability

The Python scripts used for KuNG Fu database curation are available at Zenodo (10.5281/zenodo.3996125)^52^. The source code is available at https://github.com/BadSeby/KuNG-FU-Database.
